# Feasibility, acceptability, and fidelity of Physical Activity Routines After Stroke (PARAS): a multifaceted behaviour change intervention targeting free-living physical activity and sedentary behaviour in community-dwelling adult stroke survivors

**DOI:** 10.1186/s40814-022-01139-4

**Published:** 2022-09-03

**Authors:** Sarah A. Moore, Darren Flynn, Susan Jones, Christopher I. M. Price, Leah Avery

**Affiliations:** 1 Stroke Research Group, Newcastle University, Newcastle upon Tyne, NE2 4HH UK; 2 Stroke Northumbria, Northumbria Healthcare NHS Foundation Trust, North Shields, Tyne and Wear, NE29 8NH UK; 3 Faculty of Health and Life Sciences, Northumbria University, Newcastle upon Tyne, NE1 8ST UK; 4 Centre for Rehabilitation, School of Health & Life Sciences, Teesside University, Middlesbrough, TS1 3BX UK

**Keywords:** Stroke, Physical activity, Sedentary behaviour, Healthcare professional, Behaviour change intervention, Feasibility study

## Abstract

**Background:**

Low levels of habitual physical activity and high levels of sedentary behaviour are commonly observed post-stroke. We aimed to assess the feasibility, acceptability and fidelity of a multifaceted, theory- and evidence-informed supported self-management intervention targeting physical activity and sedentary behaviour after stroke: Physical Activity Routines After Stroke (PARAS).

**Methods:**

Adult stroke survivors and healthcare professionals were recruited from North East England stroke services. Stroke survivor physical activity and sedentary behaviour were targeted by a self-management behavioural intervention supported by healthcare professionals trained in intervention delivery. The main outcomes were protocol and intervention acceptability and feasibility and fidelity of intervention delivery.

**Results:**

Eleven healthcare professionals (9 physiotherapists; 2 occupational therapists) participated in the study. Stroke survivor recruitment was lower than anticipated (19 versus target of up to 35). The healthcare professional training programme was feasible, with fidelity assessment of delivery supporting this finding. Data completeness was acceptable according to a priori criteria (>60%), except for stroke survivor questionnaire return rate (59%) and interview uptake (52%). No serious adverse events occurred. Healthcare professionals and stroke survivors perceived intervention delivery to be feasible and acceptable with minor modifications highlighted including the potential for earlier delivery in the stroke pathway.

**Conclusions:**

The study protocol and intervention delivery were feasible and acceptable to stroke survivors and healthcare professionals with modifications required before large-scale evaluation.

**Trial registration:**

ISRCTN35516780. Registered on October 24, 2018

**Supplementary Information:**

The online version contains supplementary material available at 10.1186/s40814-022-01139-4.

## Key messages about feasibility

- The feasibility of delivering a multifaceted behaviour change intervention supporting stroke survivors to engage in habitual physical activity and reduce sedentary behaviour over the long-term has not been established.
- The key feasibility findings of this study are that the study protocol and intervention delivery were feasible and acceptable to stroke survivors and healthcare professionals.
- Implications of study findings for the main study design are minor intervention modifications are required including streamlining processes and the potential for earlier delivery in the stroke pathway, and minor protocol amendments are required including improved methods for questionnaire data collection.

## Background

Physical inactivity and high levels of sedentary behaviour are common after stroke, regardless of disability [1, 2]. Targeting improvements in these behaviours can improve health outcomes and quality of life and reduce mortality [3–5]. How to optimally support stroke survivors to engage in habitual physical activity, and reduce sedentary behaviour over the long-term, has yet to be established [6]. Promising components of interventions targeting physical activity after stroke have been identified; however, a lack of explicit reference to theory and incomplete descriptions of active intervention ingredients impede replication and implementation [7].

We previously conducted multi-phase intervention development work to identify the optimal mode, form and content of an intervention targeting physical activity and sedentary behaviour after stroke [8]. This involved the application of a structured development process in accordance with the Medical Research Council (MRC) Framework for the Development and Evaluation of Complex Interventions [7, 9]. The developmental process included a systematic review [6], qualitative focus group study and co-design work with healthcare professionals (HCPs), stroke survivors and informal carers. Intervention mapping was used to guide the stages of development [10]. This approach has been used to develop interventions that target physical activity behaviour in a range of long-term health conditions [11]. Our previous development work culminated in a prototype multi-faceted intervention: Physical Activity Routines After Stroke (PARAS). The aim of the current study was to determine the feasibility of the study protocol and the feasibility and acceptability of the PARAS intervention to inform amendments prior to conducting a larger scale evaluation, if appropriate.

### Study objectives

The study objectives were (1) to assess the feasibility of delivering the study protocol in terms of recruitment and retention, HCP training programme feasibility and fidelity of intervention delivery, data completeness and patient safety and (2) to determine the feasibility and acceptability of PARAS intervention delivery from the perspectives of stroke survivors and healthcare professionals.

## Materials and methods

Specific details of the PARAS study protocol have been described previously [12] and are summarised below.

### Study design

A multi-centre, single arm, feasibility study was conducted. No changes were made to the methodology reported in our study protocol [12] prior to or after study commencement. A favourable ethical opinion was granted by North East-Tyne and Wear South Research Ethics Committee (Ref 18/NE/0255). The study was conducted in accordance with the Declaration of Helsinki. The study was registered on 24/10/2018 (ISRCTN35516780).

### Participants

#### Eligibility criteria

##### HCPs

HCPs working in community or outpatient stroke NHS rehabilitation services, who were willing to undertake PARAS intervention training and delivery and complete study outcome assessments, were eligible to participate.

##### Stroke survivors

Adult stroke survivors receiving community or outpatient rehabilitation in the study catchment area with agreed (either by stroke survivor or HCP) capacity, capability and likely benefit from a supported self-management programme targeting physical activity or sedentary behaviour were eligible. Stroke survivors who had contraindications to physical activity or had been advised by their GP or consultant to avoid increasing their physical activity levels, were excluded.

### Study setting

The setting of the study was in NHS community and outpatient stroke services in the North East of England.

#### Identification and consent

##### HCPs

Three stroke rehabilitation departments in the North East of England were approached by the research team to participate. Members of the research team visited each department providing a verbal overview of the study and written information. Following the meetings, the departments discussed which members of their teams would be appropriately placed/willing to take part in the study. The individual HCPs identified were each given an information sheet and asked to provide informed written consent to participate.

##### Stroke survivors

Stroke survivors were identified by members of the stroke rehabilitation teams or clinical trial officers who discussed the study and provided an information sheet. A minimum of 24 h was given to consider participation and ask questions before written informed consent was obtained.

### Sample size

The sample size was selected to allow adequate intervention testing within the constraints of local resources. We aimed to recruit up to 35 stroke survivors with reference to published guidelines on sample size for feasibility studies [13] and up to 12 HCPs.

### PARAS intervention

PARAS is a theory and evidence-informed multi-faceted intervention, targeting HCP consultation behaviour and stroke survivor physical activity and sedentary behaviour. Intervention mapping was used as a framework for intervention development and is described in detail in a previously published paper [8]. The APEASE criterion: affordability, practicability, effectiveness and cost-effectiveness, acceptability, side effects/safety and equity [14] were applied to the intervention design to facilitate intervention implementation.

#### HCP component

The HCP component of the PARAS intervention targeted consultation behaviour via face-to-face training, a manual and provision of one-to-one feedback on intervention delivery. The face-to-face training was delivered to HCPs over one half day by SAM and DF. Training was provided detailing the study background and rationale and targeting knowledge and skills acquisition for intervention delivery. The latter involved training on the use of brief motivational techniques to support intervention delivery. These techniques included open questions, affirmation, reflection and summarising.

Training was followed-up by provision of a manual. Each HCP delivered the intervention to two stroke survivors. The study research team then listened to audio-recordings of this intervention delivery, and with reference to a checklist of intervention content, a record was made of the presence and absence of behavioural content. This record was used to provide feedback to the HCP before they delivered the intervention to the next participant.

#### Stroke survivor component

The stroke survivor component of the PARAS intervention was a theory- and- evidence informed supported self-management intervention (supported by a HCP). PARAS was delivered in community or outpatient settings, with timing of delivery, session length and frequency of HCP contacts based on individual need. A minimum frequency of intervention delivery to each participating stroke survivor was two HCP supported sessions (baseline and review), and there was no maximum number of sessions. The baseline consultation took place face-to-face, with reviews either face-to-face or by telephone.

During the baseline consultation, a HCP supported the stroke survivor to work through a number of intervention components e.g. goal-setting, action planning and coping planning. The stroke survivor was provided with a workbook to support this process. The HCP also provided the stroke survivor with several tools, selected based on individual need e.g., self-monitoring tools.

A time to conduct a follow-up review session was agreed with the HCP and stroke survivor. The aim of the session was to review goals, provide feedback and receive support to identify and overcome barriers to achieve an increase in physical activity or decrease sedentary behaviour, respectively. Brief motivational techniques were used to enable the consultation.

**Fig. 1:**
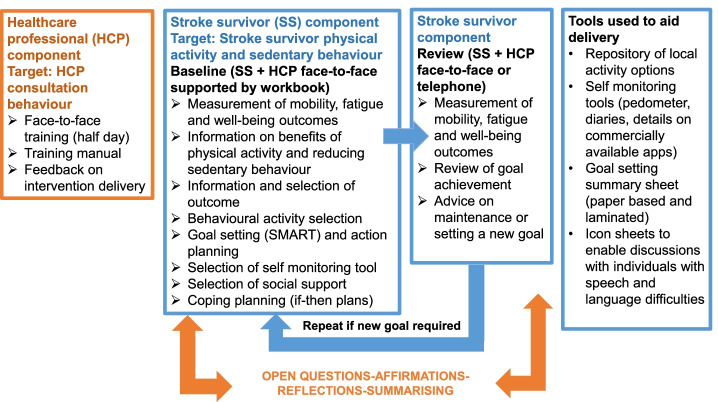
PARAS intervention components and tools

All the components and tools used in the HCP and stroke survivor components of the PARAS intervention can be viewed in Fig. 1

### Data collection

To characterise the cohort and inform a future larger scale evaluation of the PARAS intervention, the following data were collected at baseline:

#### HCPs

Sex, profession, employment status (full-time/part-time), working pattern (static/rotational), number of years qualified and number of years specialising in stroke.

#### Stroke survivors

Sex, age, occupation pre-stroke, current work status (e.g. working full-time, retired, registered sick or disabled), marital status, education, stroke type and subtype, time since stroke and any assistive device used.

To determine the feasibility of the study protocol and the feasibility and acceptability of the PARAS intervention the following data were collected:

#### Case report form

Recruitment (number of patients screened for eligibility, deemed eligible and number consented) and retention rates (number of participants completing baseline and review consultations), intervention delivery (duration of contact (minutes), time between baseline and review consultation (days), mode of intervention delivery (face-to-face or telephone), components of intervention that were personalised (e.g. self-monitoring method, type of social support, physical activity or sedentary behaviour option selected) and goal achievement (i.e. achieved target behaviour and/or outcome depending on goal). The scores for the following outcomes were collected during the baseline and review consultations: Rivermead Mobility Index [15], Warwick-Edinburgh Well-being Scale [16] and Fatigue Assessment Scale [17]. Any new medical problems or serious adverse events were recorded during appointments.

#### Questionnaire

HCPs completed a pre- and post-training questionnaire to assess attitudes and skills with regards to physical activity and sedentary behaviour post-stroke and reactions to the training. HCPs and stroke survivors both completed a post-study questionnaire on the study protocol and the PARAS intervention (See Supplementary materials Appendix C and D). Each questionnaire presented several statements that captured responses using a series of Likert scales and open questions with free text response boxes.

#### Interview

At the point of recruitment, stroke survivors were asked if they would provide their consent to an interview after receipt of the PARAS intervention. Interviews were conducted in their homes by the study lead (SAM). A semi-structured interview topic guide was used to guide the discussion (Supplementary materials, Appendix A). Following conduct of the first two interviews, the topic guide was modified, where appropriate to explore issues that emerged as salient e.g. differentiating the PARAS intervention from usual care. All interviews were audio-recorded, transcribed verbatim by a professional company and data analysed using thematic analysis [18].

#### Focus group discussions

HCPs were invited to take part in a focus group discussion at the end of the study (October 2019) to explore views on the feasibility and acceptability of PARAS. A semi-structured topic guide was used to guide discussion (Supplementary materials, Appendix B). The focus group was facilitated by the lead researcher (SAM) and two health psychologists (PM and KA).

#### Fidelity assessment

Delivery of the PARAS intervention was audio-recorded by participating HCPs to enable feedback on intervention delivery by the research team for training purposes but also to measure fidelity of delivery assessment. The audio-recordings were transcribed, and findings triangulated with written notes on intervention delivery maintained by HCPs.

#### Progression criteria

Progression criteria were set prior to study delivery in accordance with a traffic light system described by Avery et al. [19]. The criteria aimed to determine whether the intervention and outcome assessments could be delivered/conducted faithfully, with reference to the study protocol. Minor amendments to delivery indicated progression to the next stage of study with/without minor adaptation (green) moderate adaptation (amber) or significant (red) adaptation. See Table 1 for a summary of progression criteria.

**Table 1:** Study progression criteria

Criterion	Green	Amber	Red
**Study procedures**	The proposed sample size (up to 35 stroke survivors and up to 12 healthcare professionals) is achieved and retained. Study procedures are acceptable and feasible and at least 85% of outcome data is collected (i.e. case report form and questionnaire completion).	At least 14 stroke survivors and at least seven healthcare professionals recruited and retained at follow-up. Moderate changes to study procedures required (e.g. amendments to recruitment strategy and data collection procedures); and a minimum of 60% of outcome data is collected	< 14 stroke survivors and < 7 healthcare professionals recruited and retained at follow-up. Significant changes to study procedures required (e.g. eligibility criteria in order to achieve sample size; alternative data collection tools/instruments); and <60% of outcome data collected
**Intervention acceptability and feasibility**	Only minor changes to intervention required (e.g. minor changes to written information content)	Moderate changes to the intervention required (e.g. additional time required to deliver review sessions or additional intervention content is required)	Significant changes to the intervention required (e.g. change in mode of delivery, frequency of sessions and content of intervention)

### Data analysis

To determine the feasibility of the study protocol and the feasibility and acceptability of the PARAS intervention mixed methodology was applied. Quantitative and qualitative data were combined to aid interpretation of findings in relation to progression criteria. Quantitative data enabled description of study cohort and analysis compared to numerical progression criteria (e.g. recruitment rate), whereas qualitative data were used to identify themes in relation to progression criteria.

#### Quantitative analysis

Data were described and summarised using appropriate summary statistics. The distribution of nominal and ordinal data was described using frequencies and percentages. The ratio data were summarised using appropriate measures of central tendency (e.g. mean) and dispersion (standard deviation and range).

#### Qualitative analysis

Two researchers (SAM and SJ) independently read, re-read and assigned codes to the focus group transcript and the first four stroke survivor interview transcripts using a framework of a priori and emergent coding. Any disparity between coders was discussed to resolve differences in interpretation. SAM analysed the subsequent four interview transcripts, and the final interview transcript was independently analysed by both SAM and SJ. Common themes were subsequently established. Analyses of data were also discussed during regular meetings with the wider research team to identify areas for closer inspection and to enhance analysis and interpretation. Responses to open questions in questionnaires were reviewed following analyses of interview and focus group data to establish any new emergent themes. Themes (T) and subthemes (ST) were supported with verbatim quotes to provide context and enable readers to establish credibility of our findings.

#### Fidelity analysis

SAM coded all consultation transcripts to assess PARAS intervention delivery using a standardised intervention content checklist. Consultations were coded for delivery of key intervention components, including behaviour change techniques (BCTs) and brief motivational techniques. SAM completed training to identify and code BCTs using the BCT Taxonomy Version 1 [20] to facilitate this process. The four coding categories were ‘not appropriate’, ‘not delivered’, ‘delivered’ (e.g. component included but skill of delivery and understanding not demonstrated), ‘delivered well’ (e.g. demonstrated understanding and skill in delivery, for example consistent and effective use of open questions by HCP throughout the consultation). Discussion within the research team facilitated resolution of any coding issues.

## Results

### Cohort characteristics

#### Healthcare professionals

HCPs were recruited from three stroke rehabilitation services across three North East NHS Trusts. Nine physiotherapists and two occupational therapists agreed to participate. Ten participating HCPs were female and one male. Eight HCPs were part-time and three were full-time workers. All but one HCP held a static post in stroke rehabilitation, as opposed to a rotational post-across different clinical areas. Time from qualification ranged from 7 to 32 years (average 16 years) and average time specialising in stroke was 13 years (range 1–26 years).

#### Stroke survivors

Table 2 provides an overview of the characteristics of the stroke survivors who received a baseline PARAS intervention consultation. All participants were able to walk, but the majority required a stick (72%) with two also requiring a wheelchair for longer distances. Scores on the Rivermead Mobility Index at baseline ranged from 7 to 15 (maximum score 15 with higher scores indicating better mobility). Only one patient was reported to have dysphasia.

**Table 2:** Stroke survivor characteristics

Characteristic	***n*** = 18
**Sex: number (*n*) (%)**	
Male	14 (78)
Female	4 (22)
**Age (years)**	
Mean (standard deviation (SD))	58 (12)
**Occupation pre stroke:*n*(%)**	
Managers	2 (11)
Professionals	6 (33)
Student	1 (5.5)
Clerical support workers	2 (11)
Service and sales workers	3 (17)
Craft and related trade workers	3 (17)
Unemployed	1 (5.5)
**Current work status:*n*(%)**	
Retired	8 (44)
Full-time paid	2 (11)
Registered sick/disabled	6 (33)
Unemployed	1 (6)
Student	1 (6)
**Marital status:*n*(%)**	
Single	9 (50)
Married/remarried	7 (39)
Divorced	2 (11)
**Education (years)**	
Mean (SD)	13 (3)
**Cerebral hemisphere affected by stroke:*n*(%)**	
Right	9 (50)
Left	6 (33)
Bilateral	1 (6)
Unable to verify	2 (11)
**Stroke type:*n*(%)**	
Ischaemic	8 (44)
Intracerebral haemorrhage	8 (44)
Unable to verify	2 (11)
**Stroke subtype:*n*(%)**	
Total anterior circulation stroke	4 (22)
Partial anterior circulation stroke	2 (11)
Lacunar stroke	7 (39)
Posterior circulation stroke	3 (17)
Unable to verify stroke subtype	2 (11)
**Time from stroke (months)**	
Mean, (SD), [range]	13, (17), [2–139]
**Rivermead Mobility Index**	
Mean, (SD), [range]	23, (8), [7–15]
**Use of stick:*n*(%)**	13 (72)

### Protocol feasibility

#### Recruitment and retention

##### HCPs

The HCP recruits undertook training between September and October 2018. None of the HCPs dropped out during the study period. However, a proportion of HCPs was more active than others in terms of intervention delivery. One HCP delivered the intervention to three stroke survivors, six to two stroke survivors, three to one stroke survivor and one HCP did not deliver the intervention at all. The latter HCP reported that this was due to difficulty differentiating between usual care procedures and the intervention to be delivered i.e. the HCP felt they already delivered elements of the PARAS intervention as part of their usual care service. Most HCPs and stroke survivors did however indicate that the PARAS intervention was different to the rehabilitation they had previously delivered or received (T5, STA, STB, Table 3). Two participating HCPs transferred job roles during the study, meaning they could no longer deliver the intervention.

**Table 3:** Qualitative themes and sub themes and exemplar quotes extracted from stroke survivor interviews and healthcare professional focus group

Themes (T)	Sub themes (ST)	Exemplar quotes
1. Recruitment procedures require optimisation to facilitate a larger scale evaluation	A. The recruitment process didn’t generate as much interest as expected	‘…We gave out quite a lot of the information leaflets, but obviously a lot of them didn't want to take part.’ (HCP2) ‘…We had quite a few people that refused to take part, didn’t we? We did ask quite a few people that didn’t want to do it.’ (HCP3) ‘…I had one guy that just said, “I don’t want to be a guinea pig.’ (HCP5)
	B. HCP time, commitment and confidence was a barrier to recruitment	‘…I think it was time. I think there were certain periods of time where we were that busy that we didn’t really have the time to do PARAS. (HCP3) ‘…Maybe how we sold the study maybe not very well or we just asked them if they wanted to be part.’ (HCP4) ‘…Being scrutinised doing something new…you’re not going to be comfortable anyway’ (HCP1) [use of a Dictaphone recording intervention delivery]
	C. HCPs selected patients based on level of function and motivation	‘…We had a lady that we thought it would be good for but we just knew she wouldn’t engage in it. It just wouldn’t have worked with her….. Maybe we should have put her into this. We thought about it but we just thought, “Oh no, it’s not going to work.” (HCP3) ‘…I found that out of all of the patients that could be potentials, it would be the higher-level ones that I’d pick every time to engage with something like this, just to be able to read, communicate, probably physically a bit more able as well. I don’t know why.’ (HCP7) ‘…I mean really we probably should have chosen those patients who perhaps are more housebound…. I suppose you worry that if somebody looks at that benefits thing and wants all those benefits and then you’re going to do sit to stand every couple of hours, you think, “Is that going to make them feel that they’re getting any of those benefits?” (HCP4)
2. PARAS provided HCPs with new strategies to target increased physical activity with patients	A. PARAS aided development of behavioural strategies but additional training on motivation strategies for those more impaired would be beneficial	‘…I think one of the benefits I’ve had and I use it with people more than probably I ever have really considered, it was what are the barriers.’ (HCP3) ‘…I think it would be handy to have more training on the psychology side of things a little bit, the questioning and motivating patients because we definitely did shy away from the odd patient that we thought, “Well they’re not going to engage in it,” but how to engage those patients that you really can’t engage.’ (HCP3) ‘…I did find it difficult to guide patients through the process who were less mobile.’ (HCP9)
	B. Feedback on intervention delivery was reported to be useful	[feedback from study team] ‘…I think that’s useful because when you’re doing it, you’re on your own so you don’t quite know how you’re doing. So I think just to get some feedback how people are doing.’ (HCP3) ‘…The feedback that you sent was really useful just to get an idea of that. I think just to prompt you to remind you that it’s going on, you see PARAS.’ (HCP3) ‘…Yes, it was helpful to have it, yes. I mean I think like anything, it’s something that’s new. I think my feedback, there were quite a few things that I could have done differently but I needed to know that because otherwise you don’t know if you’re doing the right thing or not.’ (HCP3)
	C. Recording intervention delivery negatively affected confidence of some HCPs	‘…I don’t think the Dictaphone made everybody as relaxed as they could be…..you’ve got that pressure on yourself whereas normally you’d go in, you’d have a chat, you do your goal setting and it would all flow quite naturally.’ (HCP8) ‘…I think it’s a bit cringe knowing that your conversation is being recorded. It was always in the back of your mind, “I need to turn it on. It’s being recorded,” and you maybe weren’t as, I wasn’t as relaxed as I might have been because I knew you’d be listening.’ (HCP3)
3. The PARAS intervention is acceptable but requires minor modifications to improve delivery	A. PARAS delivery time could be reduced by providing patients with preparatory information	‘…I think it took a long time.’ (HCP6) ‘…I think probably the three assessment tools at the beginning take quite a bit of the time.’ (HCP5) ‘…Something I wondered about whether or not it would save a little bit of time is giving the patients the information before. You know all the information about the benefits of exercise and the outcomes, giving them that to look at before because it felt a little bit like they were pressured into reading all this information.’ (HCP3)
	B. Delivery of PARAS might be more appropriate earlier in the care pathway for some patients	‘...If we have this as an inpatient from rehab then that could follow, there’d be a little journey, rehab journey or something that could follow them out into the community and their goals would change from month to month.’ (HCP5) ‘...I think from the physio in hospital actually, from that early stage to when they’re going to send you home and for the early discharge team to work with you, that would be good.’ (NTP05, M, 58yrs) ‘…Timing earlier….’yes… I think when you first come out of hospital. Yes. I think that would have been better than the physiotherapist coming in here and saying, “Right, get up. We’re going to do this, we’re going to do that,” and you weren’t ready for that.’ (STP02, M, 64yrs)
	C. HCP support is a critical part of intervention delivery	‘…I think it was about right.’ (NTP05, M, 58yrs) ‘…I found it very helpful. She explained it well enough.’ (STP02, M, 64yrs) ‘…Yeah, you know, just keeping you right really on how to go.’ (NTP01, M, 67)
	D. The components of the PARAS intervention worked well together and were useful and motivational	‘...Yes, it [the workbook] just helps you to focus on what you want to gain.’ (NTP05, M, 58yrs) ‘...There was plenty of information. It helped me to pick at what was feasible for me to do and what wasn’t, what was too far.’ (STP02, M, 64yrs) ‘...I found this [the physical activity repository] really useful.’ (HCP7) ‘...Yes, just having it all in one place and just being able to flick through because you can use it for other patients as well.’ (HCP7) ‘…So I can look back at the earlier documentation and see how I have improved. So that is motivational [self-monitoring tools].’ (NTP05, M, 58yrs) ‘…As soon as I gave him the chart and the pedometer, he was off. He really enjoyed the measuring things, having a chart and having it written down. That motivated him a lot which I was stunned at because we couldn’t get him to do anything before that.’ (HCP1)
	E. Self-monitoring of physical activity for stroke survivors is challenging due to gait impairments and a lack of stroke specific tools	‘...It really comes to the speed that your legs are moving that’s the problem because I think these are really designed for more sporty people who are doing a lot of movement and fairly quick movement, whereas most people in our situation are doing slow movement.’ (SRP01, M, 63yrs) ‘...The only really major set-back that we had was the pedometers won’t work on me. so I think it’s a gait thing… It’s a bit disappointing because you’re thinking that would be a great way of judging how well you’re doing and actually how far.’ (STP01, M, 58yrs) ‘…If that was on an app, that would mean that you would see your progress, instantly and you could probably map it. It if it was possible to do that, yes it would be good.’ (NTP05, M, 58yrs)
4. PARAS was reported to aid motivation, mood, self-regulation and physical activity.	A. Improvements in physical activity were reported in relation to goals set during PARAS	‘…But walking and things like that have improved now, done obviously a lot more exercise and movement as a result of the study compared to what I was doing before, which I think is the ultimate aim’ (SRP01, M, 63yrs) ‘...When I first started walking, I got as far as the bridge, this side of the bridge and came back again and I was completely shattered and now I can … that’s not even the start of my walk.’ (STP01, M, 58yrs) ‘…So I have increased the distance quite a lot and to begin with, I was only doing about 200 steps, well I say 200 because I only count up one side, I count on my left side, so that’s 400 steps, which is about 100 metres, roughly. Now I can do a lot more than that.’ (NTP05, M, 58yrs)
	B. PARAS was reported to aid motivation and mood	‘...So I think it is motivational in that way and it does improve your mood. (NTP05, M, 58yrs) ‘...Yes, because just sitting here watching television, it’s depressing. So just getting out and being more active instead of just sitting here.’ (STP02, M, 64yrs) ‘...The fact that you’re actually applying yourself to something, rather than just sat here, it’s a bit extreme to say what I’m going to say, without a purpose, if you like.’ (STP01, M, 58yrs)
	C. PARAS facilitated development of self-regulatory skills	‘... I think it’s really helpful keeping track of what I do each day and really reflecting on how I can improve that.’ (NTP05, M, 58yrs) ‘...It’s given me an incentive to do it and an ability to measure how you’re getting on, I think that’s important, if you can measure it, I think that makes a big difference in making you push yourself.’ (STP01, M, 58yrs) ‘…It’s more when you’re out and about that you’ve got to find ways round things. This sort of thing makes you tend to look at things like that and you’ve got to try and find alternative methods of doing things.’ (SRP01, M, 63yrs)
	D. PARAS aided understanding of stroke recovery	‘...It was to go fishing but that never happened because I need two hands for that. That will never happen. …. It’s not going to happen, not for a long time anyway, not for the foreseeable future.… But I’m getting round to the fact that I’ll never go back to what I used to do. That’s never going to help me. I’ll just resent myself of the fact that that will never happen. Like I say, I take it a day at a time and just try and find what to do.’ (STP02, M, 64yrs) ‘…I mean I do know it’s going to be a long slog and I’m not going to get everything back but that’s just being realistic and then you have to say, well as long as you know that, start off with that and then think, well I’m going to get as much as I can back and if I get to the end and I think, I can’t get anything else back now, that’s it, I’m just going to go back to normal, I’m going to forget about trying to get further and further, if you like.’ (STP01, M, 58yrs) ‘…Well, I raised the bar a bit high initially – I said half a mile, but now I felt, half a mile you canny beat, so I come down to quarter of a mile.’ (SRP05, M, 69yrs)
5. The PARAS intervention was perceived to be different from usual care	A. PARAS promotes person-centred goal setting, as opposed to therapist-led goal setting	‘...[physio prior to PARAS] I felt they were trying to set their goals. They weren’t asking me what goals I had. They didn’t want to ask me that. They were just coming in, “Right, we’ll do this today. Right, we’ll take you outside.’ (STP02, M, 64yrs) ‘...When I did this study with her, it was a different type of thing. It was to change the outlook from just getting up out of the chair, bending your knee, stamping your feet a few times to then achieving other things.’ (SRP05, M, 64yrs) ‘...I think it was more them setting the goals. That was very different.’ (HCP6) ‘…We’re busy giving them a prescription, aren’t we? “This is your exercise what you should do,” rather than, like you say, listening a bit more to find out actually what do they really want to do so I think it’s good but this even helps further just to ask those open questions and listen and clarify things.’ (HCP6)
	B. Supported self-management processes applied in PARAS potentially reduce reliance on health services	‘...It’s more sustainable, isn’t it, if someone is coming up with the idea themselves and they’re self-monitoring.’ (HCP1) ‘…Patients can learn skills to set their own goals after discharge and have the resilience to prepare for barriers and setbacks.’ (HCP 9) ‘…I think if you looked at it longer term if they were to keep it up, it would potentially prevent re-referral into services if they’ve taken the ownership to do it themselves.’ (HCP3)

##### Stroke survivors

Recruitment of stroke survivors took place between 18th October 2018 and 16th October 2019. Twenty-nine patients were screened against study eligibility criteria, were eligible and were approached to take part. Nineteen stroke survivors provided consent to take part in the study. One participant was recruited but did not receive the intervention as the HCP who was supporting the stroke survivor moved to a different clinical area. Of the 18 stroke survivors who received the baseline intervention consultation, only one did not complete the intervention (e.g. met goals and did not require further PARAS support from the HCP). This stroke survivor withdrew from the study during the baseline consultation as the questioning during the Warwick-Edinburgh Mental Well-Being assessment led to her becoming upset and not wishing to continue.

HCPs at each NHS trust indicated prior to study commencement that recruitment of up to five stroke survivor participants by each HCP was feasible. However, recruitment was subsequently reported as more difficult than expected (see Table 3, Theme (T) 1 and subtheme (ST) A, for supporting direct quotes). Reasons reported included, patients declining to participate, study procedures i.e. whether the study was discussed/promoted effectively to the patients by HCPs, and the time and commitment required by HCPs to recruit patients and deliver the intervention (T1, STB, Table 3). Data also indicated that HCPs’ felt uncomfortable having their sessions recorded and offered the intervention to people they perceived may engage/benefit from the intervention e.g. higher level, more motivated patients (T1, STC, Table 3). HCP questionnaire results also revealed that two HCPs changed job role during the study limiting their ability to recruit patients and deliver the PARAS intervention.

#### HCP training programme feasibility and fidelity of delivery

Eleven HCPs completed face-to-face training before delivering the PARAS intervention. Feedback on intervention delivery was provided after HCPs had delivered the intervention to two stroke survivors, therefore only seven HCPs received feedback.

Findings from the HCP pre- and post-training attitudes, skills and reactions questionnaires, and views on the study protocol are presented in Supplementary materials Appendix C. All HCPs reported enjoying the training, 9/10 responders (90%) reported that it provided them with intervention delivery knowledge and skills, and 7/10 (70%) reported they would recommend the training to other HCPs (30% were undecided). During focus group discussion, HCPs reported the training was ‘good’ and ‘useful’ and increased their consultation skills (T2, STA, Table 3). They reported that additional training on how to use open questions and motivate/engage stroke survivors, particularly those with more marked impairment, would have been useful (T2, STA, Table 3). Feedback received on PARAS intervention delivery was reported to improve delivery (T2, STB, Table 3); however, most HCPs were uncomfortable audio-recording their consultations (T2, STC, Table 3).

Receipt of training and delivery of PARAS led to improvements in self-reported knowledge and skills development in two key areas: brief motivational techniques, including the use of open questions, and how to support identification of barriers and problem-solving in a constructive meaningful way (T2, STA, Table 3).

#### Fidelity of intervention delivery

Findings relating to delivery of intervention components and behaviour change techniques (data generated from audio-recordings intervention delivery) are presented in Table 4. In summary, most intervention components were ‘delivered’ or ‘delivered well’ according to pre-specified criteria (see the “Materials and methods” section). Only one HCP recruited the required number of participants to receive feedback and act on the feedback provided whilst supporting another stroke survivor participant. Following the receipt of feedback, elements that were ‘not delivered’ or were ‘delivered’ rather than ‘delivered well’, were improved upon, with all intervention components being ‘well delivered’.

**Table 4:** Fidelity of delivery of intervention components across stroke survivor participants where audio-recordings were available (*n*=17)

Component/behaviour change technique	Not appropriate Number of participants (%)	Not delivered Number of participants (%)	Delivered Number of participants (%)	Delivered well Number of participants (%)
**Intervention components delivery**				
*Introduction to PARAS*	1 (6)		10 (59)	6 (35)
*General advice*	1 (6)	8 (47)	2 (12)	6 (35)
*Assessment of psychological well-being, mobility and fatigue*	1 (6)		4 (24)	12 (70)
*Discussion on benefits of moving more sitting less*	1 (6)		11 (65)	5 (29)
*Discussion on patient centred outcome*	1 (6)		11 (65)	5 (29)
*Selection of PA activity*			6 (35)	11 (65)
*Identification of a specific goal*			6 (35)	11 (65)
*Identification of a method of measurement*			7 (41)	10 (59)
*Use of confidence ruler*	1 (6)	4 (24)	6 (35)	6 (35)
*Discussion on goal relevance*	1 (6)		5 (29)	11 (65)
*Identification of a timeline*			3 (18)	14 (82)
*Action planning: when, where, how, when and with whom*			7 (41)	10 (59)
*Barrier identification*			5 (29)	12 (71)
**Review**				
*Review of psychological well-being, mobility and fatigue*	2 (12)		3 (18)	12 (70)
*Review of goal achievement*	1 (6)		5 (29)	11 (65)
*Monitoring emotional consequences of goal achievement*	2 (12)		4 (24)	11 (65)
*Plan for maintenance*	12 (70)	1 (6)	1 (6)	3 (18)
**Behaviour change techniques**				
*Information about health consequences*	1 (6)		7 (41)	9 (53)
*Salience of consequences*	1 (6)		7 (41)	9 (53)
*Social support (unspecified)*		2 (12)	4 (24)	11 (65)
*Instruction on how to perform behaviour*			3 (18)	14 (82)
*Demonstration of behaviour*	17 (100)			
*Goal setting (behaviour)*			7 (41)	10 (59)
*Problem solving*			5 (29)	12 (70)
*Information about antecedents*	1 (6)		5 (29)	11 (65)
*Monitoring of emotional consequences*			4 (24)	13 (76)
*Information about social and environmental consequences*	1 (6)		3 (18)	13 (76)
*Social reward*	1 (6)		4 (24)	12 (70)
*Habit formation*			4 (24)	13 (76)
*Credible source*				17 (100)
*Action planning*			5 (29)	12 (70)
*Feedback on behaviour*	1 (6)		5 (29)	11 (65)
*Self-monitoring of behaviour*			6 (35)	11 (65)
**Delivery of brief motivational interviewing techniques**				
*Open ended questions*			8 (47)	9 (53)
*Affirmation*			5 (29)	12 (70)
*Reflective listening*			8 (47)	9 (53)
*Summarising*			4 (24)	13 (76)

#### Data completeness

##### Case-report forms (CRFs)

There were no missing data on CRFs at baseline. At review, all CRF data were complete except for scores for Rivermead Mobility Index; Warwick-Edinburgh Well-being Scale and Fatigue Assessment Scale for one stroke survivor which were left blank with no explanation provided.

##### Questionnaires, interviews, and focus group

All participating HCPs completed pre-training questionnaires 11/11 (100%), 10/11 (91%) completed post-training questionnaires and 9/11 (82%) completed study feedback questionnaires. Eight of the eleven (73%) HCPs took part in the focus group discussion and one HCP who could not attend emailed feedback for the focus group. Ten of the seventeen (59%) stroke survivors completed the post-study questionnaire and 9/17 (52%) took part in interviews.

##### Fidelity assessment

Audio-recordings of HCPs delivery of the intervention baseline consultation were conducted for 16/18 participating stroke survivors and 17/18 were conducted for review sessions. Missing recordings were due to one HCP accidently not recording the baseline consultation, and another chose not to record any session because they felt it would impact on therapeutic relations.

#### Safety

No serious adverse events were recorded. Two adverse events were recorded between baseline and review sessions. One participant developed a pressure area on their heel due to an ill-fitting ankle foot orthosis (AFO) (the HCP advised the participant to pause activity, the AFO was reviewed and replaced by an orthotist, and after the pressure area healed, the participant went on to achieve their goal). A second participant visited an accident and emergency department but was not admitted and this was unrelated to the intervention.

### Feasibility of PARAS intervention delivery

The characteristics of the delivery of the PARAS stroke survivor intervention are presented in Table 5. Walking was the most frequently selected physical activity when setting behavioural goals. None of the stroke survivors opted to target a reduction in sedentary behaviour.

**Table 5:** PARAS stroke survivor intervention delivery characteristics

Intervention delivery characteristic	
**HCP intervention contact time (minutes)**	
Mean, (Standard deviation (SD)), [range]	
*Total (baseline and review sessions)*	108 (46), [39–238]
*Baseline (n=18)*	67, (33), [24–165]
*Review (n=17)*	41, (18), [10–73]
**Time between baseline and final review (days)**	
Mean, (SD), [range]	48, (27) [21–119]
**Delivery mode: Number (*n*) (%)**	
*Baseline (n=18)*	
Face-to-face	18 (100)
Telephone	0 (0)
*Review (n=17)*	
Face-to-face	17 (100)
Telephone	0 (0)
**Self-monitoring mode:*n*(%)**	
Pedometer and diary	6 (33)
Pedometer and app	1 (6)
Pedometer, app and diary	1 (6)
Diary	3 (17)
Diary and app	1 (6)
Diary and activity tracker	4 (22)
App	2 (11)
**Social support:*n*(%)**	
Spouse	7 (38.5)
Friend	7 (38.5)
Carer	1 (6)
Partner	2 (11)
No support	1 (6)
**PA option selected:*n*(%)**	
Walking	9 (50)
Cycling	1 (6)
Home exercise programme	1 (6)
Tai Chi	2 (11)
Golf	1 (6)
Swimming	2 (11)
Gym programme	1 (6)
Housework	1 (6)
Sedentary behaviour	0 (0)
**Goal achieved at first review**	
Yes	14 (82)
No	3 (18)

Questionnaire responses indicated that most intervention components were feasible to deliver i.e. delivery of intervention in line with the protocol and within usual care (supplementary materials, Appendix C and D). However, findings generated from the qualitative data highlighted modifications would be beneficial. Focus group data indicated that although HCPs believed the intervention was beneficial for patients, the delivery was time-consuming and required streamlining to enable routine delivery (T3, STA, Table 3). To streamline the process, HCPs suggested providing stroke survivors with some of the information prior to the baseline consultation (T3, STA, Table 3). They also suggested having the option of delivering PARAS earlier in the care pathway, for example during inpatient care, to enable the development of early self-management skills. This suggestion was supported by participating stroke survivors (T3, STB, Table 3).

#### HCP support

Questionnaire data (Supplementary materials, Appendix D) confirmed that all stroke survivors (100%) believed they received good HCP support. This finding was supported by interview data (T3, STC, Table 3). However, it was difficult to extrapolate whether this finding was in relation to PARAS, usual care received, or a combination of both i.e. whether inclusion of the PARAS intervention into usual care increased satisfaction with the level of support received.

#### Social support

Most stroke survivor participants were able to identify appropriate social support from family members and friends to achieve their behavioural goals. The exception was one participant who reported having no support as he lived with elderly parents and was diagnosed with agoraphobia.

#### Workbook and repository of physical activity options

Questionnaire findings indicated that 8/10 (80%) of participating stroke survivors found the workbook useful (Supplementary materials, Appendix D), and this finding was supported by interview data (T3, STD, Table 3). HCPs reported the PARAS activity repository providing local physical activity options supported delivery of the intervention (T2, STD, Table 3). Following delivery of the intervention to a stroke survivor with limited community mobility, one HCP suggested the need for more training and tools with which to target sedentary behaviour and for home-based activities.

#### Self-monitoring tools

The most common self-monitoring method selected was a pedometer and recording steps in a diary. Of the eight participants who selected a pedometer at baseline, four subsequently selected an alternative method of self-monitoring due to the pedometer not registering steps accurately. Of those four participants, two swapped to an app or an activity tracker but still reported problems with accuracy. Stroke survivors indicated stroke-related gait impairments were the reason for the inaccuracy of the pedometers and activity trackers (T3, STE, Table 3). Despite these problems, overall, the stroke survivors reported finding the self-monitoring tools motivational (T3, STD, Table 3). Two HCPs and one stroke survivor discussed the potential for an app to record goals, progress towards goals and self-monitoring (T3, STE, Table 3), but overall approaches used were acceptable. As no goals set by stroke survivors targeted sedentary behaviour, we could not determine feasibility of methods of monitoring this behaviour.

### Intervention acceptability

Stroke survivors viewed the PARAS intervention positively with 50% of participants agreeing and 50% strongly agreeing (50%) they would recommend PARAS to others (Supplementary materials, Appendix D). Ninety percent of questionnaire respondents agreed/strongly agreed that they were more physically active or engaged in less sedentary behaviour as a consequence of taking part in PARAS. This finding was supported by interview and focus group findings (T4, STA, Table 3). In addition to qualitative data supporting increased physical activity, participants also reported several positive psychological benefits including increased motivation, mood and focus, and a sense of achievement (T4, STB, Table 3). Improvements in well-being reported during interviews were consistent with improvements on the WEMWBS where baseline mean average score increased from 49 (standard deviation (SD) = 13, range 24 to 66) to 53 (SD = 13, range 28 to 70). Stroke survivors reported that PARAS led to the development of self-regulatory skills including behavioural goal setting, coping planning and self-monitoring (T4, STC, Table 3). They also reported a greater understanding of their stroke recovery and potential which was associated with achievement or lack of achievement of goals set during the PARAS intervention (T4, STD, Table 3).

Interview data indicated that stroke survivors perceived a difference in the care they had received while taking part in the PARAS intervention. Several stroke survivors specifically reported that the goal-setting process used in PARAS was more person-centred than the approach used during usual care (T5, STA, Table 3). HCPs also reported a perceived difference between PARAS and the approach they used pre-PARAS in terms of the use of open questioning rather than prescription, and the application of behaviour change techniques not used before, such as coping planning (T5, STB, Table 3). HCPs considered that the supported self-management approach offered by PARAS could potentially reduce reliance on health services increasing sustainability (T5, STB, Table 3). Six of the nine HCP respondents stated they would like to continue to use PARAS and would recommend it to other HCPs.

### Progression criteria

Findings predominantly met the ‘amber’ (amend) progression criteria (see Table 1 for criteria). Stroke survivor and HCP recruitment and retention met the amber criteria (i.e. at least 14 stroke survivors and at least 7 HCP recruited and retained). Outcome data collection met the green (i.e. a minimum of 85% of data collected) or amber criteria (i.e. a minimum of 60% of data collected), except for completion of stroke survivor questionnaires where outcome data collection fell just below the criteria threshold (59 versus 60%). Intervention acceptability and feasibility met the amber criteria (i.e. moderate changes to the intervention required), with more intervention content required to target sedentary behaviour, streamlining of delivery and the suggestion that the intervention could be delivered earlier in the care pathway. Although amendments are required, the intervention and associated study procedures were considered feasible to deliver.

## Discussion

This study demonstrates that stroke survivors and HCPs perceive the PARAS intervention to be feasible and acceptable. Training received by HCPs delivering PARAS was well received and led to intervention components being delivered with good levels of fidelity. Findings suggest that modifications are required to refine study and intervention delivery before progression to the next stage of evaluation.

Stroke survivor participants were recruited predominantly by the HCPs who were delivering the intervention. Qualitative data indicated that HCPs were potentially selecting patients who they felt would participate and be most appropriate, rather than offering the intervention to all eligible patients. This ‘paternalistic approach’ is frequently observed in intervention research [21] and often means that those who could have benefited most were not invited to take part. Asking individuals other than those delivering the PARAS intervention to identify eligible stroke survivors may facilitate a more representative sample in a larger-scale evaluation and importantly enables all those who are eligible an opportunity to take part. Selection bias should therefore be addressed with future training ahead of intervention delivery The way in which the study was introduced/promoted to patients was also considered a recruitment barrier. This has been identified in previous stroke research highlighting a training need [22].

Other factors which may have impacted upon recruitment were the amount of time it took to deliver the intervention in addition to usual care and HCPs feeling obliged to take part in the study/training as their department had agreed to be a study site. Feeling compelled to take part in the study may also have impacted on the delivery of the intervention by HCPs. Audio-recording of intervention delivery was intended as a feedback mechanism to facilitate optimal intervention delivery and for measuring fidelity [23]. Findings, however, indicate that audio-recording impacted on recruitment, specifically selection of stroke survivors and recruitment overall. This is an important consideration when planning a future study i.e. alternative ways of assessing fidelity of delivery should be considered, and selection bias should be addressed in training delivered to HCPs.

Although the sample of stroke survivors was small, those who did participate indicated PARAS was beneficial in terms of providing them with the knowledge, motivation and self-regulation skills to increase their levels of physical activity with minimal/no side effects. This is an important early finding considering the importance of physical activity for cardiovascular and brain health after stroke [24]. With the high incidence of stroke recurrence (3-year cumulative risk score 6–25% [25]), modifying known stroke risk factors with relatively low-risk lifestyle interventions, such as physical activity, is a critically important component of stroke rehabilitation. In addition to improvements in physical activity, a range of psychological benefits were reported by stroke survivors, and these included an improvement in mood. Depression and anxiety disorders occur in up to half of stroke survivors during the first year after stroke [26]. With limited evidence on interventions to effectively target mental health [27], this is an important finding worthy of further exploration.

Although the PARAS intervention was designed to target both physical activity and sedentary behaviour, all participants elected to focus on increasing physical activity behaviour. As sedentary behaviour is a common problem following stroke [2, 28], associated with poor health outcomes [29] and reduced quality of life [30], this finding was somewhat surprising. A possible explanation is the HCPs felt more competent when promoting and supporting increased physical activity and potentially lacked the skills and confidence to effectively target sedentary behaviour. Previous research has reported that HCPs require further training to support stroke survivors to reduce sedentary behaviour [31] and stroke survivors have reported limited understanding of the health risks associated with sedentary behaviour [32]. The findings indicate there should be further iterative and co-development of PARAS intervention tools and training resources for targeting sedentary behaviour. This would enable stroke survivors to make a more informed, preference-based decision to change their physical activity and/or sedentary behaviour.

Self-monitoring of physical activity behaviour was reported to be an important active ingredient of the PARAS intervention, increasing motivation and confidence for engaging in physical activity. These findings are consistent with those reported in other interventional research in long-term health conditions [33] and highlight the importance of self-monitoring for behavioural change. The study findings, however, also emphasised the importance of selecting an appropriate self-monitoring device for each individual. Problems were identified with the accuracy of pedometers and activity trackers with individuals with altered gait, which participants reported was disappointing. This finding was perhaps not surprising, considering previous literature indicating the inaccuracy of pedometers in stroke survivors with low gait speed [34]. However, pedometers have been used successfully in stroke rehabilitation [35] and general population studies [36], and some participants do find them a helpful, relatively low-cost self-monitoring tool, indicating in the right circumstance they can be a useful tool. However, careful selection of the most appropriate self-monitoring tool for each individual is paramount to support behaviour change in the stroke population.

Although self-efficacy and outcome expectations are reported to be high in community dwelling stroke survivors, self-regulation and social support have been found to be infrequently used in the context of post-stroke recovery [37]. The incorporation of behaviour change techniques to support self-regulation and social support was shown to be important within PARAS, which is consistent with other similar small-scale studies in stroke [38–40]. However, large-scale efficacy trials have not yet confirmed this finding.

Although PARAS led to perceived benefits, several modifications to the intervention and its delivery were suggested. HCPs reported that delivery required streamlining, suggesting the inclusion of a preparation stage where stroke survivors are provided with the workbook and preparatory tasks, prior to the first consultation. With clinical services already stretched to deliver adequate levels of stroke rehabilitation [41], streamlining delivery and training, without losing potentially effective and acceptable components of the intervention, is imperative.

Although the delivery of PARAS was reported to be time-consuming, HCPs believed investing time initially on supporting self-management was worthwhile. They perceived supporting self-management could potentially reduce re-referral to stroke rehabilitation services and hospital readmission rates. Self-management capability has been found to be associated with lower health care utilisation in individuals with long-term conditions [42], supporting this finding. However, implementing this form of intervention into practice was shown to be complex in this study and further development of strategies for implementing complex interventions into rehabilitation need to be developed [43].

Our qualitative work indicated that both stroke survivors and HCPs believed that PARAS should be delivered earlier in the stroke pathway (e.g. during inpatient care), where appropriate. HCPs believed that it would facilitate reflection on physical activity progression and stroke survivors felt it would enable early development of positive behaviours. Early delivery could potentially reduce acute/sub-acute deconditioning [44], with the introduction of early self-management skills expediating recovery through increasing opportunity for intensity of practice [45]. It could also reduce deterioration in function often observed on discharge from rehabilitation particularly with older adults [46]. However, changing behaviour may be complex during inpatient stay when length of stay is short and with behaviour change potentially being more complex when the patient is not in their own environment. Alternatively, patients could be supported to use specific behaviour change techniques while in hospital and advised to adapt these for use at home.

HCPs found the training intervention beneficial, a finding supported by the high levels of fidelity of delivery observed. Audio-recording intervention delivery facilitated assessment of fidelity of delivery and receipt and is a strength of the PARAS study with rehabilitation research frequently criticised for lack of fidelity assessment [47]. However, some HCPs were reluctant to be recorded potentially impacting recruitment and delivery of the intervention and upscaling this labour-intensive process and provision of feedback to large groups of HCPs would be a significant challenge. Alternative fidelity assessment methodologies should therefore be considered during a future large-scale evaluation. 

In terms of data collection, strategies to increase the completion of questionnaires and uptake of interviews are required ahead of a larger-scale evaluation. Telephone reminders and repeat mailing strategies may facilitate questionnaire return rate and interview uptake [48] or having the option of an online questionnaire alongside the paper-based version may increase return rate. Such strategies have demonstrated to be effective in the context of other health research [49]. In addition to the baseline measures collected, a measure of cognitive impairment would be useful help determine alongside other measures already inlcuded the type of patients who may benefit from this intervention.

## Conclusion

PARAS demonstrated to be feasible and acceptable to both HCPs and stroke survivors and led to perceived benefit in terms of physical activity and well-being, but not sedentary behaviour. Fidelity of delivery was good, suggesting that the training delivered to HCPs met its objectives. Several modifications to improve PARAS were suggested by participants, including the introduction of a preparatory stage to reduce delivery time; potential to deliver the intervention earlier in the stroke pathway (i.e. during inpatient care), provision of more detailed guidance on appropriate use of self-monitoring tools and additional training for HCPs on how to support those with low levels of motivation or high levels of impairment and how to specifically target sedentary behaviour. Modifications required to improve study procedures included training to reduce recruitment selection bias and strategies to facilitate a higher response rate to the stroke survivor questionnaires and interviews. Identification of an alternative approach to fidelity assessment would also be appropriate to facilitate scalability.

## Supplementary Information

**Additional file 1: Appendix A.** Stroke survivor interview guide. **Appendix B.** HCP focus group discussion guide. **Appendix C.** HCP pre- and post-training and protocol questionnaire Likert scores. **Appendix D.** Stroke survivor post intervention questionnaire Likert scores.

## Data Availability

The datasets used and/or analysed during the current study are available from the corresponding author on reasonable request.
