# Supplementary material for: Feasibility, acceptability, and fidelity of Physical Activity Routines After Stroke (PARAS): a multifaceted behaviour change intervention targeting free-living physical activity and sedentary behaviour in community-dwelling adult stroke survivors
Source: Pilot Feasibility Stud. 2022 Sep 3;8:197. doi: 10.1186/s40814-022-01139-4 (PMC9440503; doi:10.1186/s40814-022-01139-4)
Supplement: Supplementary file 1 — Additional file 1: Appendix A. Stroke survivor interview guide. Appendix B. HCP focus group discussion guide. Appendix C. HCP pre- and post-training and protocol questionnaire Likert scores. Appendix D. Stroke survivor post intervention questionnaire Likert scores. [file 40814_2022_1139_MOESM1_ESM.docx]

**Supplementary materials**

**Appendix A** Stroke survivor interview guide

*Stroke survivor interview guide*

1. **Firstly, please could you tell me a little about…**
   - How you found out about the PARAS study?
   - What other (if any) support did you receive from the community stroke team?
2. **Due to taking part in the PARAS study what types of things did you learn?**
   - Benefits of moving more / sitting/laying down less
   - Options available for moving more / sitting/laying down less
   - How to develop goals and plans for moving more / sitting/laying down less
3. **What do you find helpful about the PARAS study?**
   - Support from the healthcare professional [stroke community team)
   - The workbook – if yes, which parts?
     1. Was there too much information? Was there anything that was not clear?
   - The tools to help you record how much you were moving and sitting/laying down?
   - Anything else you found helpful
4. **What has been the impact of the PARAS study on you and/or your family?**
   - Do you move more and /or sit/lay down less?
   - Any changes to the types of activities you do regularly (or could not do after your stroke)?
   - Any changes to your health and/or wellbeing (e.g. confidence, stress and anxiety, enjoyment of life, sense of control over your life, something else)?
   - Any changes to your family relationships, social life, or other aspects of your life?
5. **Is there anything you found unhelpful about the PARAS study?**
   - Is there anything that could be done to improve the PARAS study?
   - What other information/support would have been helpful to you?
6. **Would you recommend the PARAS study to other stroke survivors?**
7. **Anything we have not discussed that you feel is important related to the PARAS study more generally?**

**Appendix B** HCP focus group discussion guide

1. **As an introductory question - What is the present situation like (encourage stroke survivors to increase and decrease their levels of physical activity and sedentary behaviour respectively) without the PARAS intervention?**
2. **How did PARAS influence the way you typically talk to stroke survivors about physical activity/sedentary behaviour?**
   - How does PARAS differ to other projects providing support to adult stroke survivors in the community?
3. **Were there any benefits of PARAS for stroke survivors?**
4. **Were there any benefits of PARAS for you as healthcare professionals?**
5. **What worked well with the PARAS intervention?**
6. **Did you make use of all the intervention components?**
   - Workbook
   - Laminated discussion cards
   - Goal setting – behaviour
   - Goal setting - outcomes
   - Action planning
   - Coping planning
   - Self-monitoring tools
   - Booklet of physical activity resources
7. **What worked not so well with the PARAS intervention?**
   - Is there anything that could be done to improve the PARAS intervention?
   - What other information/support would have been helpful?
8. **Could you provide some specific examples of how PARAS has benefitted stroke survivors and their families?**
   - Moving more and /or sitting/laying down less?
   - Changes to the types of activities they do regularly (or could not do after your stroke)?
   - Any changes to their health and/or wellbeing (e.g. confidence, stress and anxiety, enjoyment of life, sense of control over your life, something else)?
   - Any changes to their family relationships, social life, or other aspects of your life?
9. **In your view what are the barriers and enablers to use of PARAS in the current stroke community service?**
10. **Is there anything that we have not discussed, that you feel is important?**

**Appendix C** HCP pre- and post-training and protocol questionnaire Likert scores

|  | **Pre-training (responders 11/11)** | | | | | **Post-training (responders 10/11)** | | | | |
| --- | --- | --- | --- | --- | --- | --- | --- | --- | --- | --- |
|  | **Strongly**  **Disagree** | **Disagree** | **Undecided** | **Agree** | **Strongly**  **Agree** | **Strongly**  **Disagree** | **Disagree** | **Undecided or N/A** | **Agree** | **Strongly**  **Agree** |
| ***Attitudes*** | | | | | | | | | | |
| *1.It is important to support stroke survivors to engage in free-living physical activity* |  |  |  | 2 (18) | 9 (82) |  |  | 1 (10) | 1 (10) | 8 (80) |
| *2.It is important to support stroke survivors to reduce the time they sit and lay down* |  |  | 1 (9) | 4 (36) | 6 (55) |  |  | 1 (10) | 2 (20) | 7 (70) |
| *3.It is my role to support stroke survivors to engage in long-term free living physical activity and reduce sedentary time.* |  |  |  | 5 (45) | 6 (55) |  |  |  | 2 (20) | 8 (80) |
| *4.There are benefits to stroke survivors engaging in free-living physical activity and reducing sedentary behaviour* |  |  |  | 2 (18) | 9 (82) |  |  |  | 1 (10) | 9 (90) |
| **Skills** | | | | | | | | | | |
| *1.I am aware of the research evidence supporting engagement in physical activity and reducing sedentary behaviour after stroke* |  |  |  | 11 (100) |  |  |  |  | 8 (80) | 2 (20) |
|  | **Strongly**  **Disagree** | **Disagree** | **Undecided** | **Agree** | **Strongly**  **Agree** | **Strongly**  **Disagree** | **Disagree** | **Undecided or N/A** | **Agree** | **Strongly**  **Agree** |
| *2.I have a good understanding of the absolute and relative contraindications to engaging in physical activity after stroke* |  | 1 (9) | 2 (18) | 7 (64) | 1 (9) |  |  |  | 8 (80) | 2 (20) |
| *3.I am confident that I can motivate stroke survivors to engage in more physical activity and reduce their sedentary behaviour* |  |  | 2 (18) | 8 (73) | 1 (9) |  |  | 1 (10) | 7 (70) | 2 (20) |
| *4.I am confident I can adapt physical activity programmes to the needs of individual stroke survivors* |  |  | 1 (9) | 8 (73) | 2 (18) |  |  |  | 6 (60) | 4 (40) |
| *5.I am confident in the use of self-report scales used in the PARAS intervention* | 1 (9) | 3 (27) | 6 (55) | 1 (9) |  |  |  | 2 (20) | 6 (60) | 2 (20) |
| *6.I am confident that I can support stroke survivors to identify their preferred physical activity/sedentary behavioural outcomes* |  | 1 (9) |  | 8 (73) | 2 (18) |  |  |  | 7 (70) | 3 (30) |
| *7.I am confident in supporting stroke survivors to set SMART goals* |  |  | 2 (18) | 7 (64) | 2 (18) |  |  |  | 8 (80) | 2 (20) |
| *8.I am confident in supporting stroke survivors to set action plans* |  |  | 2 (18) | 7 (64) | 2 (18) |  |  |  | 8 (80) | 2 (20) |
| *9.I am confident in supporting stroke survivors to identify barriers to doing more physical activity, and support the development of coping plans* |  |  | 3 (27) | 7 (64) | 1 (9) |  |  |  | 9 (90) | 1 (10) |
| *10.I am confident in using tools to help stroke survivors self-monitor their activity levels* |  | 1 (9) | 6 (55) | 3 (27) | 1 (9) |  |  |  | 8 (80) | 2 (20) |
| *11.I am confident in sign-posting stroke survivors to local physical activity options* |  | 1 (9) | 3 (27) | 6 (55) | 1 (9) |  |  |  | 8 (80) | 2 (20) |
| *12.I am confident in reviewing stroke survivors’ progress towards their physical activity/sedentary behaviour goals.* |  |  | 1 (9) | 9 (82) | 1 (9) |  |  |  | 9 (90) | 1 (10) |
| *13.I understand study processes (participant identification, eligibility criteria; consent; baseline assessment; intervention delivery; assessment).* |  | 3 (27) | 4 (36) | 4 (36) |  |  |  |  | 10 (100) |  |
| **Reaction to training (responders 10/11)** | | | | | | | | | | |
| *1.I enjoyed the face-to-face training* | N/A | N/A | N/A | N/A | N/A |  |  |  | 4 (40) | 6 (60) |
| *2.The face-to-face training provided me with the knowledge and skills I require to deliver the PARAS intervention* | N/A | N/A | N/A | N/A | N/A |  |  | 1 (10) | 4 (40) | 5 (50) |
| *3.I would recommend the training programme to other healthcare professionals* | N/A | N/A | N/A | N/A | N/A |  |  | 3 (30) | 3 (30) | 4 (40) |
| **Reaction to study protocol (responders 9/11)** | | | | | | | | | | |
| *1.The eligibility criteria for stroke survivors are suitable* | N/A | N/A | N/A | N/A | N/A |  |  |  | 7 (78) | 2 (22) |
| *2.I was able to deliver the PARAS intervention with to up to five stroke survivors during the study period* | N/A | N/A | N/A | N/A | N/A | 3 (33) | 4 (44) |  | 2 (22) |  |
| *3.The process for gaining participant consent was feasible* | N/A | N/A | N/A | N/A | N/A |  |  |  | 6 (67) | 2 (22) |
| *4.I was able to deliver the PARAS intervention in line with the protocol* | N/A | N/A | N/A | N/A | N/A |  |  |  | 8 (89) | 1 (11) |
| *5.The mode of delivery of PARAS to stroke survivors was suitable e.g. face-to-face or telephone* | N/A | N/A | N/A | N/A | N/A |  | 1 (11) | 1 (11) | 4 (44) | 3 (33) |
| *6.I was able to deliver the PARAS intervention within my usual delivery of stroke rehabilitation* | N/A | N/A | N/A | N/A | N/A |  | 4 (44) | 1 (11) | 4 (44) |  |
| *7.The workbook was a useful resource* | N/A | N/A | N/A | N/A | N/A |  | 1 (11) | 1 (11) |  | 7 (78) |
| *8.The intervention toolkit was useful (e.g. pedometers, instructions, PA diaries)* | N/A | N/A | N/A | N/A | N/A |  |  | 2 (22) | 6 (67) | 1 (11) |
| *9.The physical activity resource booklet was useful and easy to use* | N/A | N/A | N/A | N/A | N/A |  | 1 (11) | 1 (11) | 1 (11) | 6 (67) |
| *10.The healthcare professional training helped me to deliver the intervention* | N/A | N/A | N/A | N/A | N/A |  |  | 1 (11) | 5 (56) | 3 (33) |
| *11.The feedback from the audio recorded sessions of intervention delivery was useful and appropriate* |  |  |  |  |  |  |  | 4 (44) | 1 (11) | 5 (56) |
| *12.I would like to continue using the PARAS intervention in the future with stroke survivors* |  |  |  |  |  |  | 1 (11) | 2 (22) | 3 (33) | 3 (33) |
| *13. I would recommend the PARAS intervention to other healthcare professionals* |  |  |  |  |  |  |  | 3 (33) | 3 (33) | 3 (33) |

**Appendix D**  Stroke survivor post intervention questionnaire Likert scores

| **Questions** | **Number of responses and percentage**  **Responders 10/17 (59%)** | | | | |
| --- | --- | --- | --- | --- | --- |
|  | **Strongly**  **Disagree** | **Disagree** | **Undecided** | **Agree** | **Strongly**  **Agree** |
| 1. *I now move more and sit/lay down less as a result of taking part in the PARAS study* |  |  | 1 (10) | 3 (30) | 6 (60) |
| 1. *I had good support from my healthcare professional during my participation in the PARAS study* |  |  |  | 1 (10) | 9 (90) |
| 1. *I know how to set goals to help me to move more and sit/lay down less* |  |  | 1 (10) | 2 (20) | 7 (70) |
| 1. *I know how to set action plans to help me with achieving my goals for moving more and sitting/laying down less* |  |  | 1 (10) | 4 (40) | 5 (50) |
| 1. *I know how to set coping plans to help me overcome things that might get in the way of me moving more and sitting/laying down less* |  |  |  | 6 (60) | 4 (40) |
| 1. *I know how to self-monitor and record how much I move or sit/lay down on a daily basis* |  |  |  | 5 (50) | 5 (50) |
| 1. *I am aware of options for moving more / sitting less in my local area for stroke survivors* |  |  |  | 6 (60) | 4 (40) |
| 1. *The workbook was useful for helping me to move more and sit/lay down less* |  | 2 (20) |  | 2 (20) | 6 (60) |
| 1. *I would recommend the PARAS study to other stroke survivors* |  |  |  | 5 (50) | 5 (50) |
